# Supplementary material for: Identification of photoperiod-induced specific miRNAs in the adrenal glands of Sunite sheep (Ovis aries)
Source: Front Vet Sci. 2022 Jul 22;9:888207. doi: 10.3389/fvets.2022.888207 (PMC9354845; doi:10.3389/fvets.2022.888207)
Supplement: Supplementary file 1 [file Data_Sheet_1.ZIP › Supplementary file/miRDeep2 file.zip!/miRDeep2 file/Know_Structure/SP42A2/oar-mir-3959.pdf]

miRBase precursor : oar-mir-3959
Total read count : 22652
oar-miR-3959-5p read count : 19997
oar-miR-3959-3p read count : 2655
remaining reads : 0

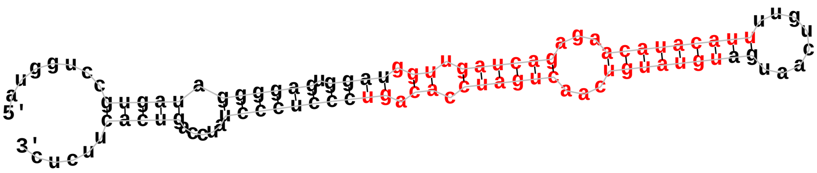

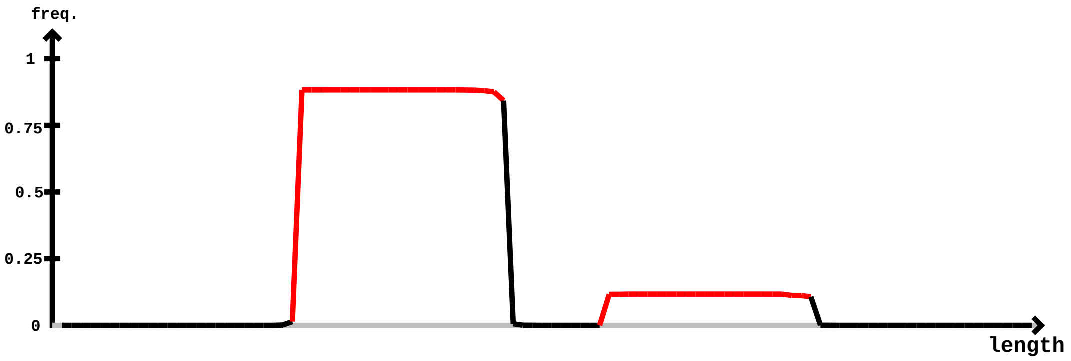

oar-miR-3959-5p

oar-miR-3959-3p

5'- augguccgugauaggggaguggaugguugaucagagaacauacauuuugucaaugauguaugucaacugauccacagucccucccuauccagucacuucuc -3'	exp		
.......(((((.((((((.((((.((.((((((...(((((((((........)))))))))...)))))).)).))))))))))).....)))))).....	reads	mm	sample
......................augguugaucagagaacau..	1	0	seq
......................augguugaucagagaacaua...	2	0	seq
......................augguugaucagagaacauac..	5	0	seq
......................augguugaucagagaacauaca...	17	0	seq
......................augguugaucagagaacauacauu...	2	0	seq
.......................ugguugaucagagaacaua...	1	0	seq
.......................ugguugaucagagaacauaca...	7	0	seq
.......................ugguugaucagagaacauacau..	24	0	seq
.......................ugguugaucagagaacauacauu...	263	0	seq
........................gguugaucagagaacau..	7	0	seq
........................gguugaucagagaacaua...	7	0	seq
........................gguugaucagagaacauac..	44	0	seq
........................gguugaucagagaacauaca...	74	0	seq
........................gguugaucagagaacauacau..	716	0	seq
........................gguugaucagagaacauacauu...	18694	0	seq
........................gguugaucagagaacauacauuu..	117	0	seq
........................gguugaucagagaacauacauuuu...	9	0	seq
........................gguugaucagagaacauacauuuug..	3	0	seq
.........................guugaucagagaacauacauu...	2	0	seq
..........................uugaucagagaacauacauuuu...	1	0	seq
...........................ugaucagagaacauacauuuugu...	1	0	seq
....uguaugucaacugaucc............................	2	0	seq
....uguaugucaacugauccac..........................	112	0	seq
....uguaugucaacugauccaca.........................	13	0	seq
....uguaugucaacugauccacag........................	106	0	seq
....uguaugucaacugauccacagu.......................	2404	0	seq
....uguaugucaacugauccacaguc......................	3	0	seq
....uaugucaacugauccacagu.......................	8	0	seq
....uaugucaacugauccacaguc......................	2	0	seq
....uaugucaacugauccacagucc.....................	5	0	seq
